# Discovery of Novel ncRNA Sequences in Multiple Genome Alignments on the Basis of Conserved and Stable Secondary Structures

**DOI:** 10.1371/journal.pone.0130200

**Published:** 2015-06-15

**Authors:** Yinghan Fu, Zhenjiang Zech Xu, Zhi J. Lu, Shan Zhao, David H. Mathews

**Affiliations:** 1 Department of Biochemistry & Biophysics and Center for RNA Biology, University of Rochester Medical Center, Rochester, New York, United States of America; 2 MOE Key Lab of Bioinformatics, School of Life Sciences, Tsinghua University, Beijing, China; 3 Department of Biostatistics & Computational Biology, University of Rochester Medical Center, Rochester, New York, United States of America; Ben-Gurion University, ISRAEL

## Abstract

Recently, non-coding RNAs (ncRNAs) have been discovered with novel functions, and it has been appreciated that there is pervasive transcription of genomes. Moreover, many novel ncRNAs are not conserved on the primary sequence level. Therefore, de novo computational ncRNA detection that is accurate and efficient is desirable. The purpose of this study is to develop a ncRNA detection method based on conservation of structure in more than two genomes. A new method called Multifind, using Multilign, was developed. Multilign predicts the common secondary structure for multiple input sequences. Multifind then uses measures of structure conservation to estimate the probability that the input sequences are a conserved ncRNA using a classification support vector machine. Multilign is based on Dynalign, which folds and aligns two sequences simultaneously using a scoring scheme that does not include sequence identity; its structure prediction quality is therefore not affected by input sequence diversity. Additionally, ensemble defect was introduced to Multifind as an additional discriminating feature that quantifies the compactness of the folding space for a sequence. Benchmarks showed Multifind performs better than RNAz and LocARNATE+RNAz, a method that uses RNAz on structure alignments generated by LocARNATE, on testing sequences extracted from the Rfam database. For de novo ncRNA discovery in three genomes, Multifind and LocARNATE+RNAz had an advantage over RNAz in low similarity regions of genome alignments. Additionally, Multifind and LocARNATE+RNAz found different subsets of known ncRNA sequences, suggesting the two approaches are complementary.

## Introduction

Traditionally, RNA was considered to simply be important in expressing proteins. The discovery of a wide range of RNA molecules that do not function as intermediates of protein translation has changed that view. RNA sequences are involved in important biological functions such as self-cleavage catalysis, post-transcription gene regulation and genome defense [1–3]. These RNA sequences that function without being translated to proteins are called non-coding RNA (ncRNA) sequences. RNA transcripts can therefore be characterized in three ways, protein-coding, non-coding, or non-functional.

The ENCODE project (“the Encyclopedia Of DNA Elements”), a research project that aims to identify all the functional elements in the human genome sequence, showed 62% of the human genome is transcribed [4]. 5% of this transcriptional output can be explained as exons by GENCODE, which aims to annotate all the gene features in the human genome [4]. Although not all of the transcripts are functional, this suggests that only a small portion of ncRNA functions are known to us, despite their importance.

One difficulty with the computational discovery of novel classes of ncRNA by comparative genomics is the low sequence conservation of ncRNAs [5]. Many functional ncRNAs, however, have conserved secondary structures [6]. Therefore, secondary structure conservation can serve as strong evidence that an RNA has function. Methods exploiting RNA secondary structure conservation have been developed, but there is room to improve the accuracy [6–10]. For example, the overlap in the sets of putative ncRNA using different methods only contains a small portion of all the predictions [11–13].

Current methods for detecting structured RNA adopt a range of strategies. RNAz [8, 14] and EvoFold [7] adopt an align-then-fold strategy. RNA secondary structure folding is performed on a multiple sequence alignment, where the input alignment is fixed. Then the predicted structure is evaluated to give a probability or a score of the candidate being ncRNA. The performance of this strategy is hindered by the alignment quality, which is adversely affected by low sequence similarity. To overcome limitations in alignment quality, CMfinder [9] searches for common structures among unaligned sequences by comparing local structures predicted on single sequences. Another approach is taken by methods based on Dynalign [10, 15] and Foldalign [16], which use algorithms that fold and align two sequences simultaneously. The structural alignment quality is therefore not adversely affected by low sequence similarity because the sequence alignment is guided by RNA secondary structure. For example, Dynalign performs better at ncRNA discovery on homologous RNA sequences with low sequence similarities than RNAz [10]. A variation on this is the LocARNATE+RNAz approach that uses LocARNATE to align sequences using secondary structure information and then RNAz to classify the sequence [14].

Although Dynalign has been successfully applied to ncRNA discovery, only two sequences can be taken as input in that method. Therefore, it cannot take advantage of the additional compensatory base pair change information provided by using more than two sequences. Multilign [17], a method based on Dynalign, can predict common secondary structures among more than two sequences by progressively building the alignment. Multilign was shown to be more accurate at structure prediction than Dynalign. In this contribution, a method called Multifind is reported to detect ncRNAs in multiple sequences using Multilign. Multiple features concerning structural conservation and stability were taken as input features to train a support vector machine. Benchmarks on known families of ncRNA taken from Rfam 10.1 [18, 19] show Multifind performs better than RNAz and LocARNATE+RNAz. For ncRNA discovery in genomes or transcriptomes, Multifind can serve as a complement to RNAz and LocARNATE+RNAz in finding ncRNAs in low identity aligned regions.

## Methods

### Structure Determination

Multilign was used to determine common structures among multiple sequences [17]. Multilign uses Dynalign, a program that folds and aligns two sequences simultaneously to find their common structures. In Multilign, Dynalign progressively constructs the consensus structure for multiple sequences. Among the input sequences, one sequence is chosen as the index sequence to participate in pairwise Dynalign calculations with each other sequence. Base pairs are only allowed in the index sequence if they are contained in a set of low free energy structures predicted by Dynalign with each other sequence. In the final iteration of refinement, Multilign folds the index sequence, where its structure is well-determined, with each other sequence with Dynalign calculations to determine the common structure.

For single-sequence structure prediction, Fold [20] and MaxExpect [21] in the RNAstructure package [22] were used. The free energy changes were calculated using the most recent nearest neighbor parameter set [20, 23, 24], with the exception that the parameter for adding an additional helix to a multibranch loop was set to -0.6 kcal/mol to be consistent with the estimate based on optical melting experiments [25].

### SVM implementation and usage

The SVM implementation LIBSVM, http://www.csie.ntu.edu.tw/~cjlin/libsvm/, was used. LIBSVM implements SVM formulations both for classification and regression analysis [26]. Each of these implementations has a set of parameters that need to be optimized. In this study, ε-support vector regression (ε-SVR) using the radial basis function (RBF) kernel was used for regression analyses. This formulation has three parameters to optimize, C, ε and γ. Classification analyses used the c-support vector classification (c-SVC) with the RBF kernel that has two parameters (C and γ) to optimize. LIBSVM provides two python scripts (grid.py and gridregression.py) that were used to optimize the parameters by searching for their optimal values in user-specified grids. Parameter values were evaluated according to 5-fold cross validation on the training data sets.

### Features for Distinguishing ncRNA

Multifind uses three features to distinguish ncRNA sequences from background sequences. These features are structural conservation index, average single sequence folding free energy Z score and average single sequence normalized ensemble defect Z score. Additionally, the average Shannon entropy for the sequence alignment provides context for the values of the three features that is important for classification accuracy.

### Structure conservation index (*SCI*)

Structural conservation index (*SCI*) quantifies the structural conservation among RNA secondary structures [8]. It is defined as:

SCI=EcES, (1)

where *E*_*c*_ is the average of the folding free energies of the structures predicted by Multilign. *E*_*s*_ is the average of the folding free energies of the structures predicted with Fold, a single sequence structure prediction tool in RNAstructure [22].

### Average single sequence folding free energy Z score

To quantify the significance of the thermodynamic stability of the structures predicted for single sequences, a Z score was used, i.e. the number of standard deviations the stability is different as compared to the mean of a suitable sample. To generate a sample to determine the background folding free energy change, the original sequence was shuffled, only maintaining the nucleotide frequency. The structures are predicted for each sequence and the Z score is then defined as:

$$Z=\frac{E-\mu }{\sigma },$$ (2)

where *E* is the folding free energy change of an individual sequence predicted on single sequence, *μ* is the average folding free energy change of the shuffled sequences and σ is the standard deviation of the folding free energy changes of the shuffled sequences.

Calculating the Z score by shuffling sequences, however, is computationally costly. SVMs were used to predict the Z scores for single sequences, as done previously [8]. First, 17,303 sequences were generated with length from 30 to 150 nucleotides, GC content from 25% to 75%, G/GC ratio from 25% to 75% and A/AU ratio from 25% to 75%. Then each sequence was shuffled 1,000 times to get the average and the standard deviation of the folding free energy changes of the shuffled sequences for each target sequence. Two separate regression SVMs were trained to predict average and standard deviation. The inputs to each SVM are GC content, G/GC ratio, A/AU ratio and sequence length.

### Average single sequence normalized ensemble defect Z score

Functional RNAs are not only constrained to fold into thermodynamically stable structures. To function, the RNA structural conformational space needs to be well constrained to one or at most a few dominant structures. Prior analysis showed natural occurring RNA sequences have well-constrained conformational spaces compared to random sequences with the same nucleotide content [27]. To describe compactness of folding space of RNAs, the distance, *d*, between two structures *s*_1_ and *s*_2_ of a sequence is defined as:

$$d(s_{1},s_{2})=N-\sum_{\begin{array}{l} 1\leq i\leq N\\ 1\leq j\leq N+1 \end{array}}S_{i,j}(s_{1})S_{i,j}(s_{2}),$$ (3)

where *i* and *j* are indexes of nucleotide position and *N* is the sequence length. *S*_*i*,*j*_(*s*_1_) = 1 if base pair *i*-*j* is in structure s_1_, and is 0 otherwise. Similarly, *S*_*i*,*N*+1_(*s*_1_) = 1 if *i* is unpaired and 0 otherwise. It is clear *d*(*s*_1_, *s*_2_) = *N* if every nucleotide in the sequence adopts a different conformation in two structures and *d*(*s*_1_,*s*_2_) = 0 if two structures are completely identical. This formulation of distance between structures is chosen for the convenience of calculating ensemble defect, as shown below. The distance of one structure, *s*, from its own thermodynamic ensemble therefore can be defined as:

$$n(s,\Omega )=\sum_{\sigma \in \Omega }p(\sigma ,\Omega )d(s,\sigma ),$$ (4)

where *Ω* is the set of all the possible structures of the sequence. *p*(*σ*,*Ω*) is the probability of structure *σ* in *Ω*, which can be obtained by calculating the partition function. It can be shown that:

$$n(s,\Omega )=N-\sum_{1\leq i\leq N}P(i,s),$$ (5)

where *P*(*i*,*s*) is the probability of nucleotide *i* adopting the specific conformation in structure *s*, i. e. the probability of nucleotide *i* being base paired with the specific nucleotide in structure s or the probability of nucleotide *i* being unpaired if *i* is unpaired in structure *s*. The term, *n*(*s*,*Ω*), is the ensemble defect of structure *s*, and *n*(*s*, *Ω*) /*N* is the normalized ensemble defect [28]. MaxExpect in RNAstructure predicts the lowest ensemble defect structure, which can be used to define *s*. For this structure, the MaxExpect score is *n*(*s*,*Ω*), when γ equals one [21].

To infer the significance of the normalized ensemble defect, SVMs were trained to predict the average normalized ensemble defect and normalized ensemble defect standard deviation of the shuffled sequences. The input sequences were the same sequence set used for determining average folding free energy change and its standard deviation for SVM training. The average ensemble defect Z score of all the sequences was included as a feature in the classification SVM.

### Shannon entropy

Although *SCI* is useful for predicting sequences that are ncRNA, when sequences have high identity, *SCI* is close to one and therefore it cannot identify ncRNAs effectively. The folding free energy change Z score and ensemble defect Z score predicted on single sequences are then more meaningful to identify ncRNAs. For the SVM to put the correct emphasis on these features, a feature that can describe the diversity of the aligned sequences, average Shannon entropy, was included [29]:

$$S=\frac{1}{N}\sum_{i\in A}S_{i},$$ (6)

where *S*_*i*_ is the Shannon entropy of one column, *A* is the set of all the columns in an alignment, *N* is the number of columns in an alignment.

$$S_{i}=-\sum_{k\in C}p_{k}\ln p_{k},$$ (7)

where *C* is all the types of characters in a RNA alignment, *C* = {A,C,G,U,-} and *p*_*k*_ is the frequency of character *k* in the column *i*. The higher the Shannon entropy, the more diverse the sequences in the alignment are.

### Machine Training and Evaluation

The four features (structural conservation index, average single sequence folding free energy Z score, average single sequence normalized ensemble defect Z score and Shannon entropy) were included in ncRNA classification training. Training data were drawn from the Rfam database 10.1 [18, 19]. All the Rfam sequence families with average lengths from 30 to 150, with over 25 members, and with conserved structures were chosen for obtaining sequences. This provided 164 families. In each family, an equal number of groups containing from 3 to 6 sequences were randomly selected with replacement. The number of sequence groups drawn from each family was proportional to the size of the family. In total, 22,308 sequence groups were drawn to constitute the positive training set. Then each group of sequences was aligned using ClustalW [30]. For each alignment generated, all the columns were randomly shuffled to build up the negative training set with the exact same size as the positive training set. The complete data set of positive and negative controls for training and testing is provided as Supporting Information (S1 and S2 Files).

SVM training was run on these alignments using the ‘-b 1’ option. This trained the SVM to output probability of an alignment being ncRNA, which provides the information needed for using a threshold for classification.

Genomic testing data were generated by cutting genome alignments into windows. Then ncRNA detection methods were run on the windows. Three genome alignments were used for benchmarking, which are (1) *Escherichia coli* (RefSeq Accesssion: NC_000913) aligned with *Salmonella typhi* (NC_004631), *Salmonella paratyphi* (NC_011147), *Shigella boydii* (NC_010658) and *Klebsiella pneumonia* (NC_011283), (2) *Streptomyces coelicolor* (NC_003888) aligned with *Streptomyces avermitilis* (NC_03155) and *Streptomyces griseus* (NC_010572) and (3) *Saccharomyces cerevisiae* (NC_001133) aligned with *Saccharomyces paradoxus*, *Saccharomyces mikatae*, *Saccharomyces kudriavzevii*, *Saccharomyces bayanus*, *Saccharomyces castellii* and *Saccharomyces kluyveri*. The sequences for the *E*. *coli* and *S*. *coelicolor* alignments were downloaded from NCBI RefSeq database [31], and both alignments were generated using the “progressiveMauve” command in Mauve [32] with no extra options other than the input sequences. The *Saccharomyces cerevisiae* alignment was downloaded from the UCSC genome browser [33]. For all the alignments, only the alignment blocks that include all the input sequences and are in intergenic regions of the *E*. *coli*, the *S*. *coelicolor* or the *S*. *cerevisiae* genome were kept for subsequent processing and analysis. The intergenic regions’ coordinates of the *E*. *coli* and the *S*. *cerevisiae* genomes were inferred from the coordinates of the genes included in the RefSeq files. The intergenic regions’ coordinates of the *S*. *coelicolor* genome were provided by Vockenhuber et al. [13]. Then all the alignment blocks were cut into 100 nt windows with 50 nt step size. Known ncRNA locations in *E*. *coli* and *S*. *coelicolor* genomes were acquired from the Rfam database 10.1 [18, 19]. Additional known ncRNAs in the *S*. *coelicolor* genome identified by deep sequencing experiments were also included [13]. ncRNA locations in *S*. *cerevisae* were acquired from the RefSeq file.

### Scoring

For ncRNA prediction, there are two criteria to evaluate the prediction: First is the fraction of the real ncRNAs detected. This is the sensitivity:

$$sensitivity=\frac{TP}{TP+FN},$$ (8)

where *TP* is the true positives (ncRNAs correctly classified as ncRNA) and *FN* is the false negatives (ncRNA incorrectly classified as not being ncRNA).

The second criterion is the fraction of the non-ncRNAs correctly classified as not ncRNA, the specificity:

$$specificity=\frac{TN}{TN+FP},$$ (9)

where *TN* is the true negatives (sequences that are not ncRNA and correctly classified as not being ncRNA) and *FP* is the false positive (sequences that are not ncRNA and correctly classified as being ncRNA). Because of the large lengths of genomes, a large number of false positives would be generated if the specificity is not high. Therefore, high specificity is critical.

## Results

### Single Sequence Free Energy Z Score Estimation

Following previous practice, the single sequence folding free energy Z scores were used in Multifind to estimate folding stability [8]. The accuracy of the Z-score estimation by SVMs was evaluated by benchmarking on randomly generated sequences. 1,000 random sequences were generated with GC content, G/GC ratio and A/AU ratio randomly picked within the range of 25% to 75%, and each sequence was shuffled 1,000 times to generate a background set of sequences. The average folding free energy change and standard deviation in folding free energy change for the background sequences were calculated and were used to determine Z scores for the 1,000 random sequences. The SVMs were also used to predict the Z scores on these sequences. The predicted Z score was plotted against sampled Z score (S1A Fig) and was shown to be highly correlated by the linear correlation coefficients (*R*_*free energy z*_ = 0.998). The correlation shows that these SVMs have high prediction accuracies for the folding free energy Z score.

### Ensemble Defect

For an RNA sequence to be functional, it needs to be able to fold into stable structures. Additionally, the number of structures it can fold into needs to be limited [27]. The ensemble defect of a secondary structure describes how different the structure is from its alternatives, weighted by the ensemble probability [28]. By predicting the structure with the minimum ensemble defect of a RNA sequence, the compactness of its conformational space can be quantified. In this study, the mean predicted minimum ensemble defect Z score of all the input sequences was taken as an input for the SVM training. The accuracy of the Z score prediction, taken over the same data set and background sequences used to evaluate the prediction accuracy of the folding free energy change Z score, is illustrated in S1B Fig (*R*_*ensemble defect z*_ = 0.999).

### ncRNA classification on Rfam dataset

The training dataset is composed of 164 families containing 22,308 real ncRNA alignments and the same number of negative control alignments, acquired by shuffling the columns of the ncRNA alignments. All the ncRNA alignments were acquired from Rfam seed alignments [18, 19]. To test the classification method, a cross-validation approach was used, with four rounds. In each round, families were randomly chosen to form a testing test, containing roughly 10% of all the alignments in the dataset. Testing sets were chosen according to family identity, i.e. a family either appeared in the training or testing set but not both, which avoided homology between the training set and testing set. The four testing sets do not overlap, therefore they are completely independent.

Receiver-operator characteristic curves (ROC curves) were plotted to demonstrate the quality of classification. These curves show the tradeoff in sensitivity (the fraction of ncRNA alignments correctly classified as ncRNA) and specificity (the fraction of shuffled alignments correctly classified as not being ncRNA) by plotting sensitivity as a function of false positive rate, i.e. 1-specificity. This is done by iterating over the probability threshold for which a sequence is classified as ncRNA. A perfect classifier would have a point in the upper-left-hand corner of the plot at 100% sensitivity and 100% specificity.

**Fig 1:**
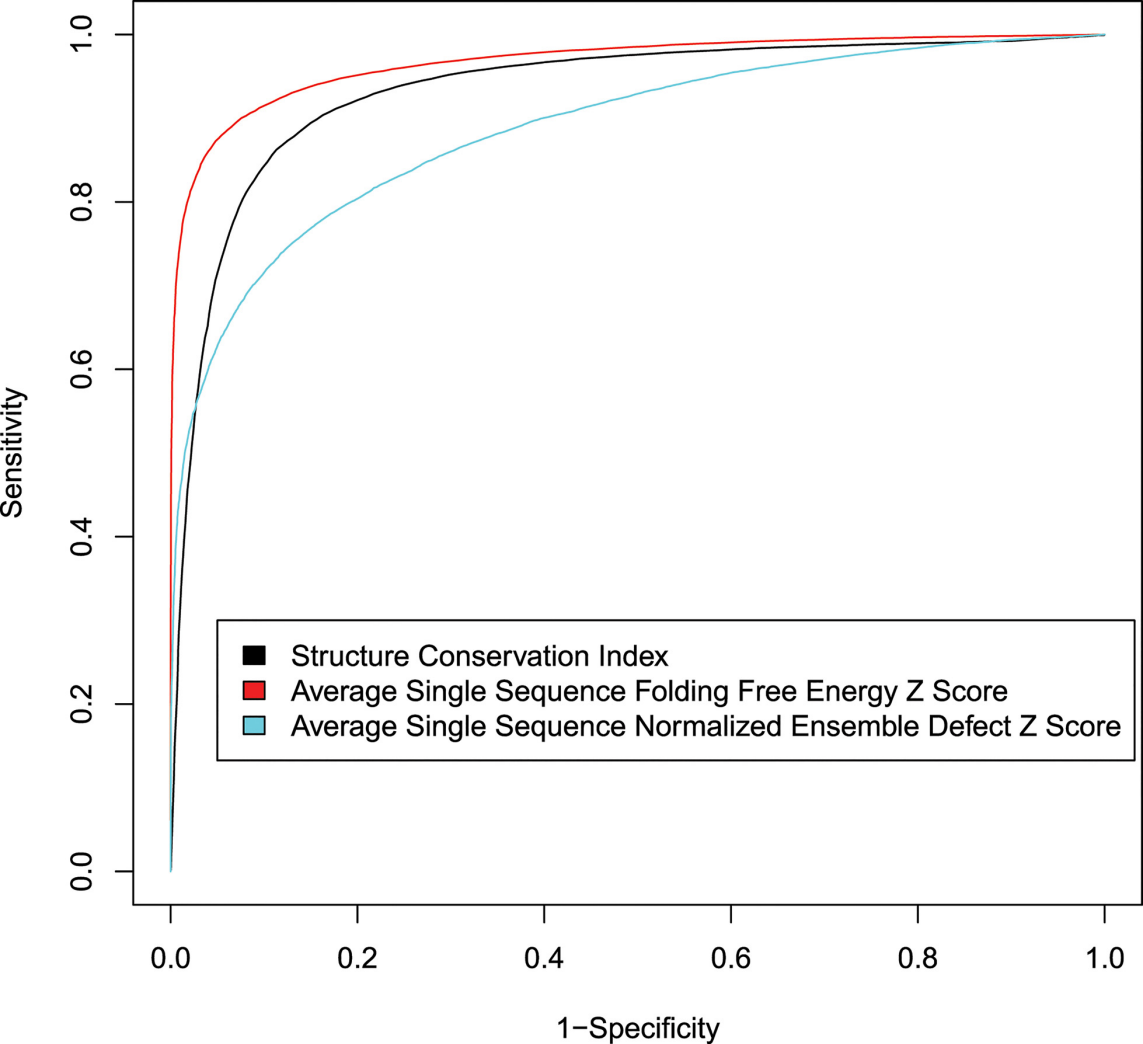
ROC curves for ncRNA discovery features. For each feature, cut offs were scanned to generate ROC curves. The hypotheses are that alignments with higher *SCI*, lower free energy Z scores and lower ensemble defect Z scores are more likely to be ncRNA. The ROC curves were generated using the entire Rfam dataset.

The three features, *SCI*, single sequence folding free energy Z score, and single sequence ensemble defect Z score provide information that can identify ncRNA as compared to sequences from shuffled genome alignments. ROC curves were plotted for each feature (Fig 1). Four SVM classification machines were trained to output classification probability using four training sets and tested on the four testing sets. RNAz [8, 14], LocARNATE+RNAz [14], Dynalign/SVM [10] and Multifind without ensemble defect Z score were also tested on the four testing sets. For the tests of Dynalign/SVM, two sequences from each alignment were randomly chosen because Dynalign/SVM is limited to two sequences as input. For RNAz calculations, the sequences were aligned using ClustalW, as done previously [8, 14], and then this alignment was used as input. For LocARNATE+RNAz [14], multiple unaligned sequences are first taken as input to LocARNATE, which then outputs a structural alignment that can serve as input for RNAz.

**Fig 2:**
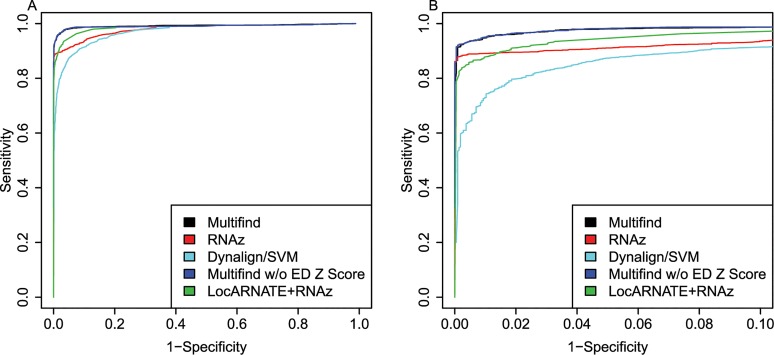
ROC curves for benchmarks on the first RFAM testing set. (A) ROC curves for Multifind, Multifind trained without ensemble defect Z score, RNAz, LocaRNATE+RNAz and Dynalign/SVM on the first testing set. (B) The high-specificity range of the ROC curves for Multifind, Multifind trained without ensemble defect Z score, RNAz, LocARNATE+RNAz and Dynalign/SVM on the first testing set.

**Fig 3:**
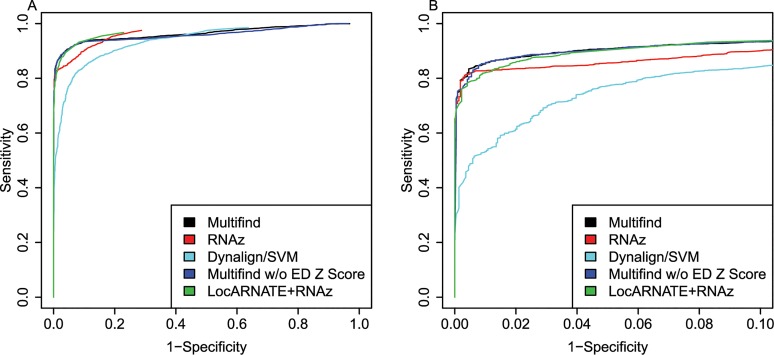
ROC curves for benchmarks on the second RFAM testing set. (A) ROC curves for Multifind, Multifind trained without ensemble defect Z score, RNAz, LocaRNATE+RNAz and Dynalign/SVM on the second testing set. (B) The high-specificity range of the ROC curves for Multifind, Multifind trained without ensemble defect Z score, RNAz, LocARNATE+RNAz and Dynalign/SVM on the second testing set.

**Fig 4:**
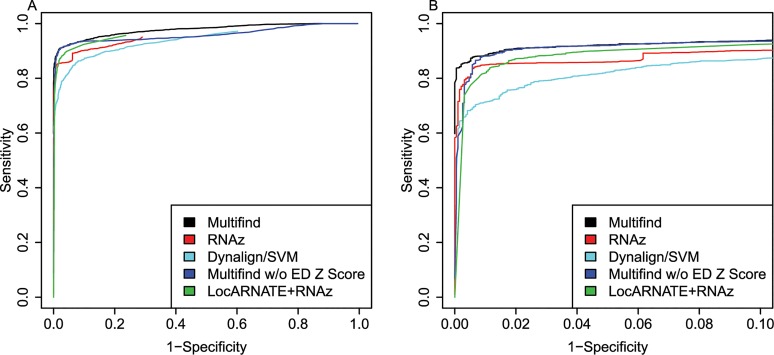
ROC curves for benchmarks on the third RFAM testing set. (A) ROC curves for Multifind, Multifind trained without ensemble defect Z score, RNAz, LocaRNATE+RNAz and Dynalign/SVM on the 3rd testing set. (B) The high-specificity range of the ROC curves for Multifind, Multifind trained without ensemble defect Z score, RNAz, LocARNATE+RNAz and Dynalign/SVM on the third testing set.

**Fig 5:**
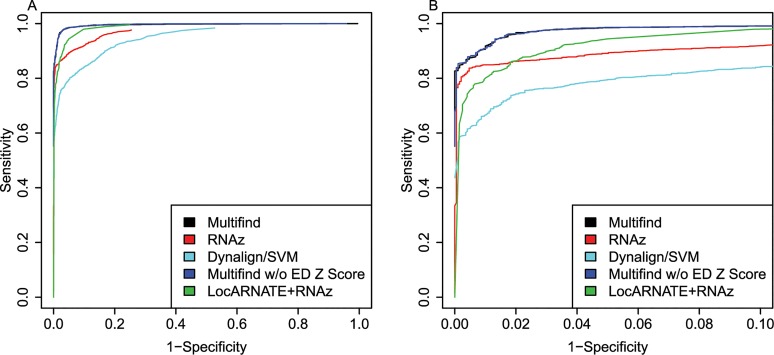
ROC curves for benchmarks on the fourth RFAM testing set. (A) ROC curves for Multifind, Multifind trained without ensemble defect Z score, RNAz, LocaRNATE+RNAz and Dynalign/SVM on the fourth testing set. (B) The high-specificity range of the ROC curves for Multifind, Multifind trained without ensemble defect Z score, RNAz, LocARNATE+RNAz and Dynalign/SVM on the fourth testing set.

ROC curves were generated for all four testing sets (Figs 2–5) and there was variability across the four sets. Multifind has higher sensitivity than RNAz and LocARNATE+RNAz at most specificities across the four testing sets. Because Dynalign/SVM can only take two sequences as input, its performance was not as good as Multifind, LocARNATE+RNAz or RNAz. For each testing set, plots were made for both all specificities and for the high-specificity regions (1-Specificity ≤ 0.10). For genome scans, the most important part of the ROC curve is the high-specificity region (Specificity > 0.98) because scans performed at low specificity would generate large numbers of false positives because of the relatively low prevalence of ncRNAs in genomes. In all sets, Multifind performed best in this high-specificity region, although RNAz performed similarly to Multifind on set two (Fig 3).

**Fig 6:**
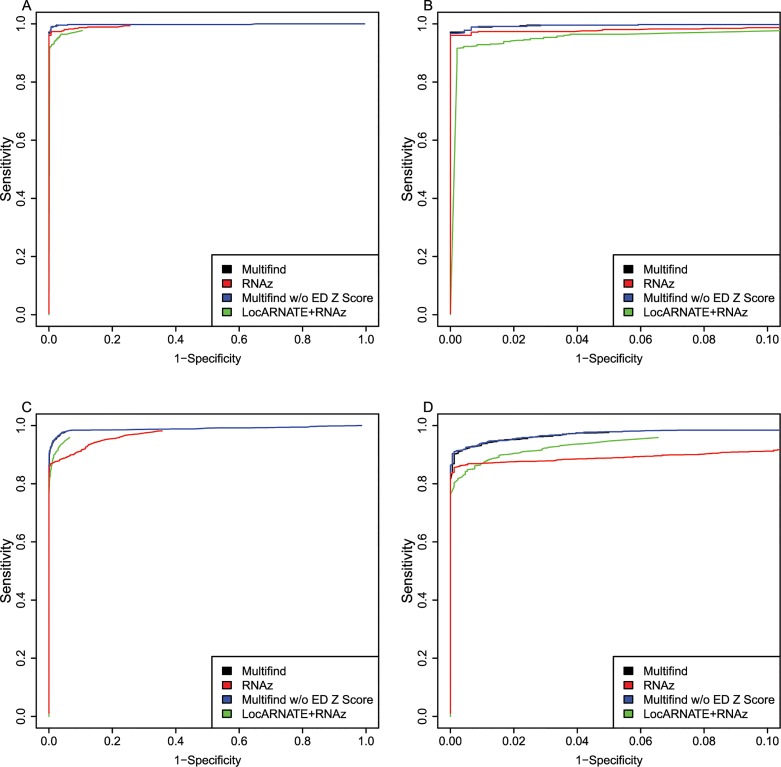
ROC curves for benchmarks on high and low entropy ranges of the first RFAM test. (A) ROC curves for Multifind, Multifind trained without ensemble defect Z score, RNAz, LocARNATE+RNAz and Dynalign/SVM on the low-entropy range (<0.3) of the first testing set. (B) The high-specificity range of the ROC curves for Multifind, Multifind trained without ensemble defect Z score, RNAz, LocARNATE+RNAz and Dynalign/SVM on the low-entropy range (<0.3) of the first testing set. (C) ROC curves for Multifind, Multifind trained without ensemble defect Z score, RNAz, LocARNATE+RNAz and Dynalign/SVM on the high-entropy range (>0.3) of the first testing set. (D) The high-specificity range of the ROC curves for Multifind, Multifind trained without ensemble defect Z score, RNAz, LocARNATE+RNAz and Dynalign/SVM on the high-entropy range (>0.3) of the first testing set.

**Fig 7:**
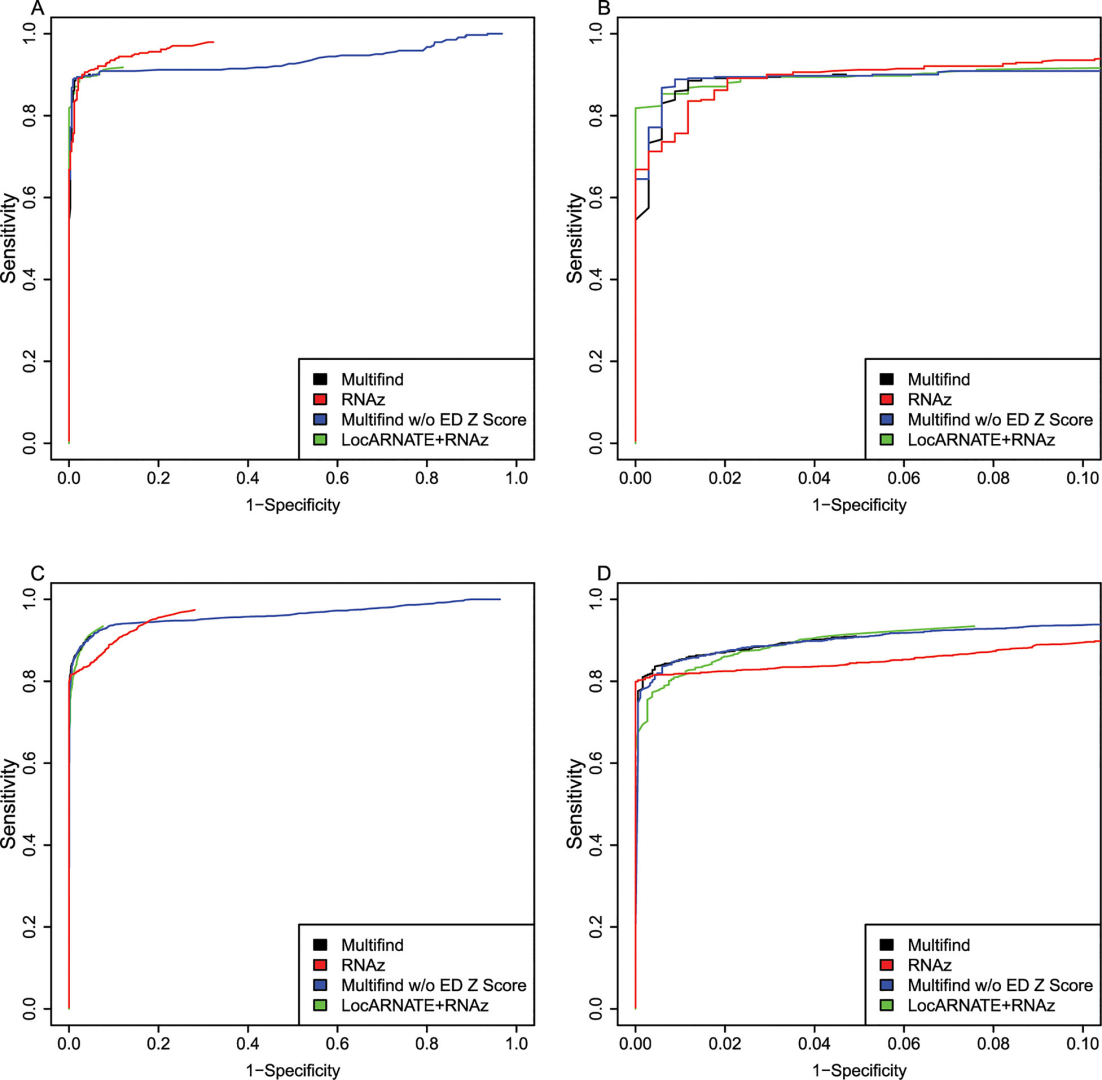
ROC curves for benchmarks on high and low entropy ranges of the second RFAM test. (A) ROC curves for Multifind, Multifind trained without ensemble defect Z score, RNAz, LocARNATE+RNAz and Dynalign/SVM on the low-entropy range (<0.3) of the second testing set. (B) The high-specificity range of the ROC curves for Multifind, Multifind trained without ensemble defect Z score, RNAz, LocARNATE+RNAz and Dynalign/SVM on the low-entropy range (<0.3) of the second testing set. (C) ROC curves for Multifind, Multifind trained without ensemble defect Z score, RNAz, LocARNATE+RNAz and Dynalign/SVM on the high-entropy range (>0.3) of the second testing set. (D) The high-specificity range of the ROC curves for Multifind, Multifind trained without ensemble defect Z score, RNAz, LocARNATE+RNAz and Dynalign/SVM on the high-entropy range (>0.3) of the second testing set.

**Fig 8:**
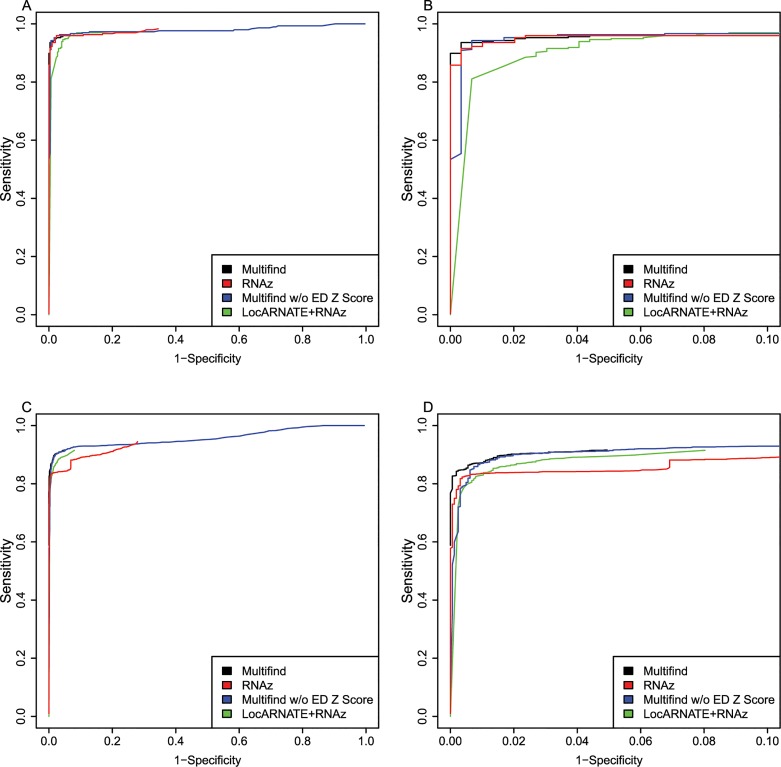
ROC curves for benchmarks on high and low entropy ranges of the third RFAM test. (A) ROC curves for Multifind, Multifind trained without ensemble defect Z score, RNAz, LocARNATE+RNAz and Dynalign/SVM on the low-entropy range (<0.3) of the third testing set. (B) The high-specificity range of the ROC curves for Multifind, Multifind trained without ensemble defect Z score, RNAz, LocARNATE+RNAz and Dynalign/SVM on the low-entropy range (<0.3) of the third testing set. (C) ROC curves for Multifind, Multifind trained without ensemble defect Z score, RNAz, LocARNATE+RNAz and Dynalign/SVM on the high-entropy range (>0.3) of the third testing set. (D) The high specificity range of the ROC curves for Multifind, Multifind trained without ensemble defect Z score, RNAz, LocARNATE+RNAz and Dynalign/SVM on the high-entropy range (>0.3) of the third testing set.

**Fig 9:**
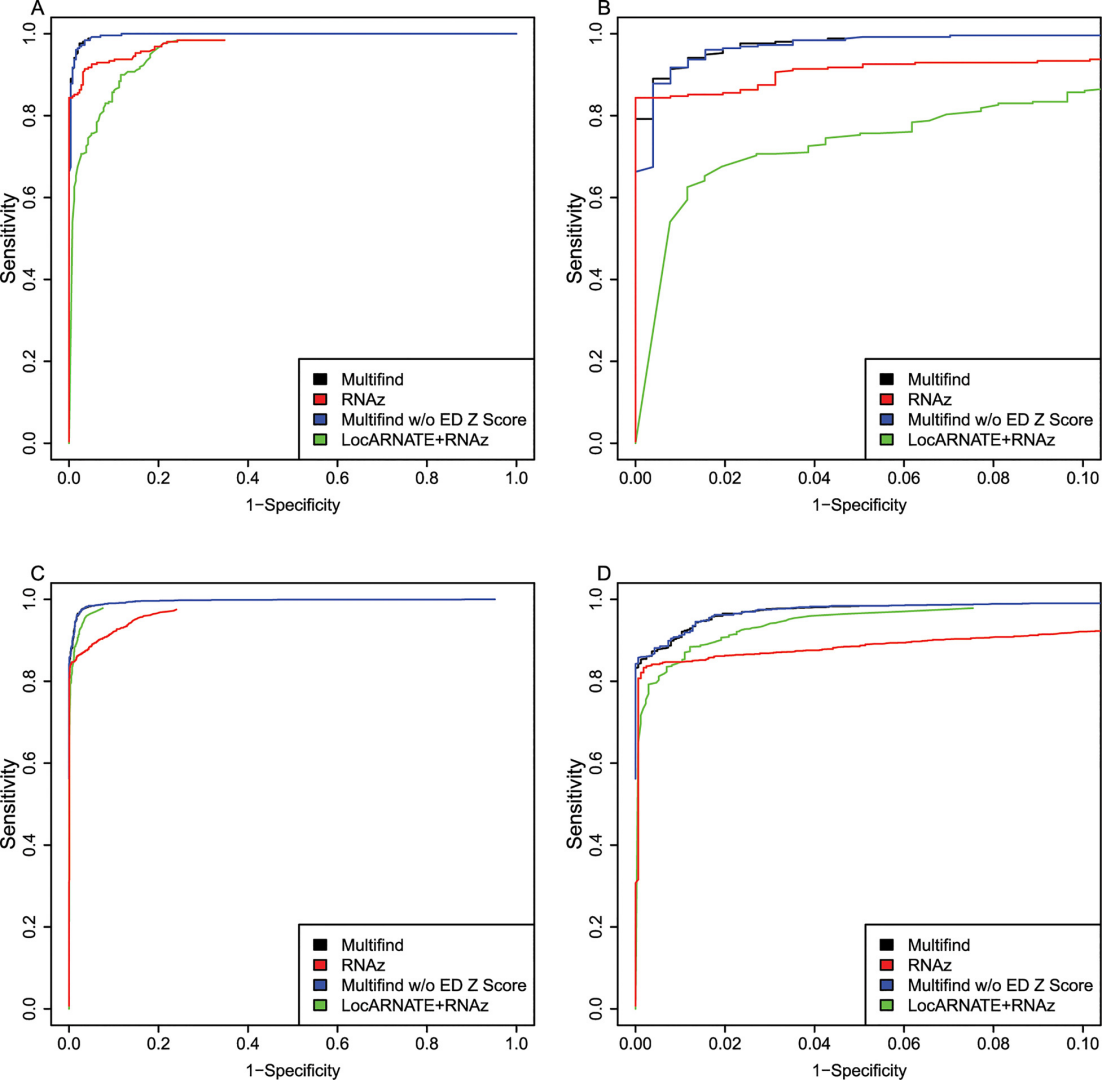
ROC curves for benchmarks on high and low entropy ranges of the fourth RFAM test. (A) ROC curves for Multifind, Multifind trained without ensemble defect Z score, RNAz, LocARNATE+RNAz and Dynalign/SVM on the low-entropy range (<0.3) of the 4th testing set. (B) The high-specificity range of the ROC curves for Multifind, Multifind trained without ensemble defect Z score, RNAz, LocARNATE+RNAz and Dynalign/SVM on the low-entropy range (<0.3) of the fourth testing set. (C) ROC curves for Multifind, Multifind trained without ensemble defect Z score, RNAz, LocARNATE+RNAz and Dynalign/SVM on the high-entropy range (>0.3) of the fourth testing set. (D) The high specificity range of the ROC curves for Multifind, Multifind trained without ensemble defect Z score, RNAz, LocARNATE+RNAz and Dynalign/SVM on the high-entropy range (>0.3) of the fourth testing set.

One hypothesis was that Multifind will perform better than RNAz on low similarity alignments because Multilign aligns and folds multiple sequences simultaneously. To test this hypothesis, each testing set was divided into two categories with Shannon entropy larger or smaller than 0.3, and the accuracies of the classifiers measured using ROC curves for each category (Figs 6–9). On testing sets with high entropy, Multifind has a distinct advantage over RNAz. The advantage of LocARNATE+RNAz over RNAz is also apparent because LocARNATE aligns and folds sequences simultaneously. In most high-entropy testing sets, Multifind also has higher sensitivity than LocARNATE+RNAz at all specificities. At the highest specificities (Specificity > 0.98), Multifind outperforms LocARNATE+RNAz in all four data sets. Figs 4 and 8 also show that Multifind has some advantage over Multifind without ensemble defect on the 3rd testing set, therefore ensemble defect can provide independent predictive power.

### ncRNA classification on genomes

The ability of Multifind to identify ncRNAs in genomes was tested against LocARNATE+RNAz and RNAz, using scans of three genomes, *Escherichia coli* [34], *Streptomyces coelicolor* [35] and *Saccharomyces cerevisiae* [36]. *E*. *coli* was aligned with four species: *Salmonella typhi* [37], *Salmonella paratyphi* [38], *Shigella boydii* and *Klebsiella pneumonia* [39]. *S*. *coelicolor* was aligned with two species: *Streptomyces avermitilis* [39] and *Streptomyces griseus* [40]. Both alignments were made using the multiple genome alignment tool Mauve [32]. A seven-way yeast alignment was downloaded from the UCSC genome browser [33], http://genome.ucsc.edu/, including: *Saccharomyces cerevisiae*, *Saccharomyces paradoxus*, *Saccharomyces mikatae*, *Saccharomyces kudriavzevii*, *Saccharomyces bayanus*, *Saccharomyces castellii* and *Saccharomyces kluyveri* [41]. The scans were restricted to non-repeat, intergenic regions. These regions were divided into 100 nt windows with 50 nt step size.

A considerable number of ncRNAs are known in these genomes. Known ncRNA locations in *E*. *coli* and *S*. *coelicolor* genomes were acquired from the Rfam database 10.1 [18, 19]. Additional ncRNAs in the *S*. *coelicolor* genome identified by deep sequencing experiments were also included [13]. ncRNA locations in *S*. *cerevisae* were acquired from the NCBI database [42]. The distribution of the lengths of the ncRNAs acquired from the above mentioned databases are provided in S1 Table. A window that has over 30% of its nucleotides overlapping with any ncRNA or which contains over 50% of nucleotides of a ncRNA was identified as a ncRNA window. The distribution of the windows in all the genome alignments according to the percentage of nucleotides that overlap with a ncRNA is provided in S2 Table.

Multifind, LocARNATE+RNAz and RNAz were applied on these windows. To evaluate the results of these three methods, instead of plotting ROC curves, plots of true positives as a function of total number of predicted candidates were used. This is because it is unknown whether unannotated regions are truly not ncRNA. The ratio between true positives and total candidates is a predicted success rate, assuming that most predicted ncRNAs not annotated as ncRNA are false positives.

**Fig 10:**
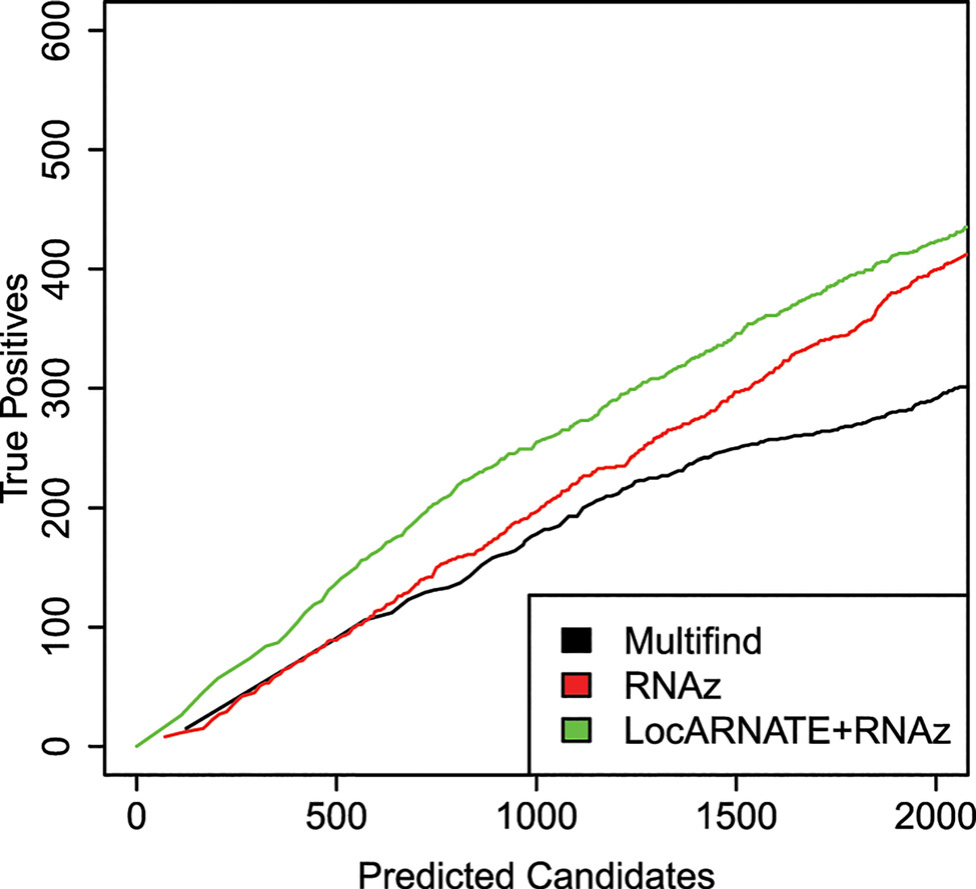
True positives versus total number of predicted candidates for the *S*. *cerevisiae* genome.

**Fig 11:**
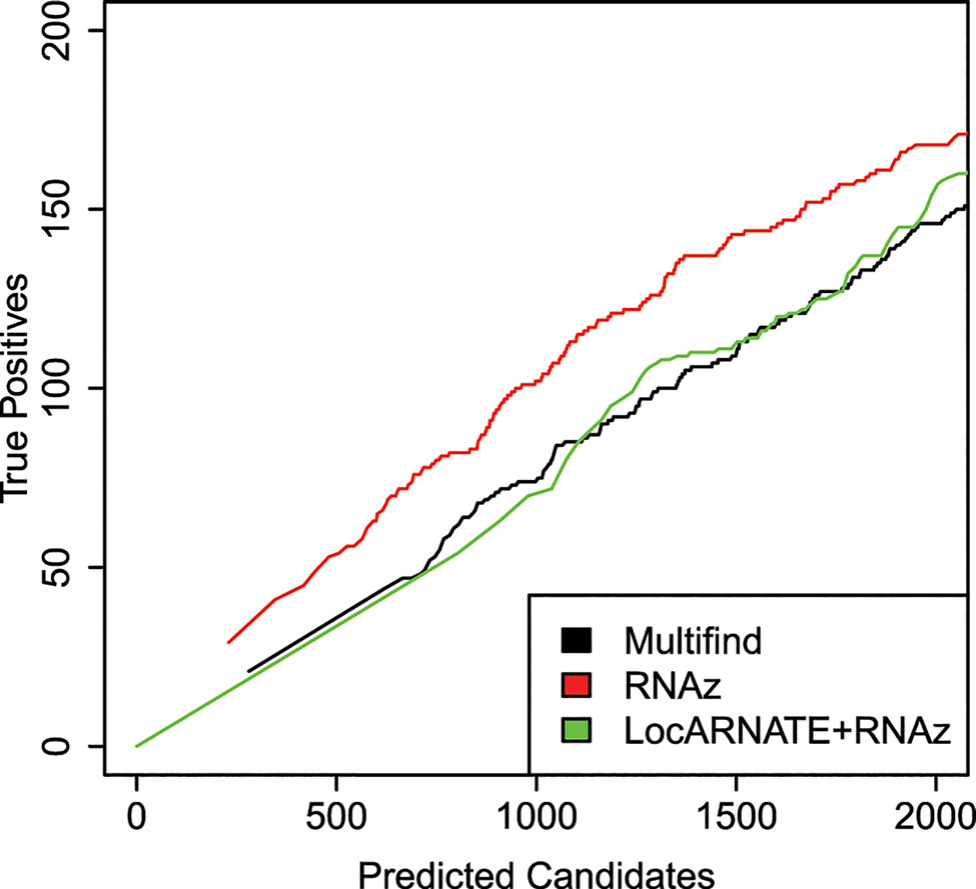
True positives versus total number of predicted candidates for the *E*. *coli* genome.

**Fig 12:**
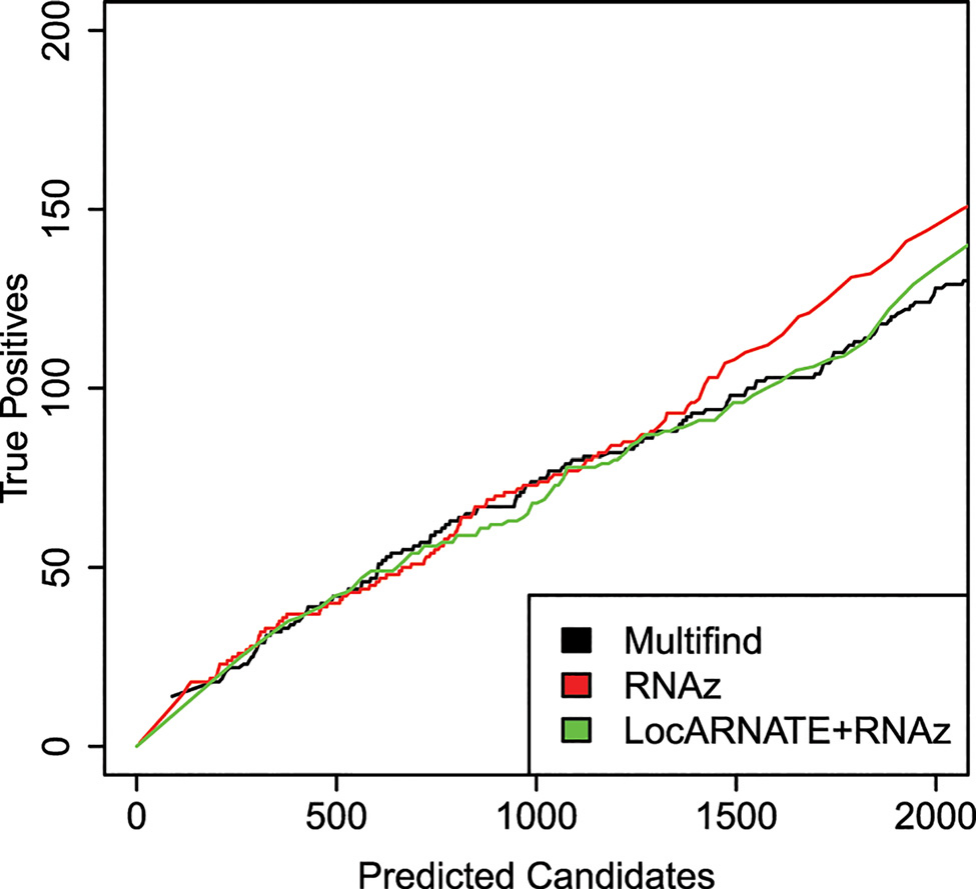
True positives versus total number of predicted candidates curve for the *S*. *coelicolor* genome.

**Fig 13:**
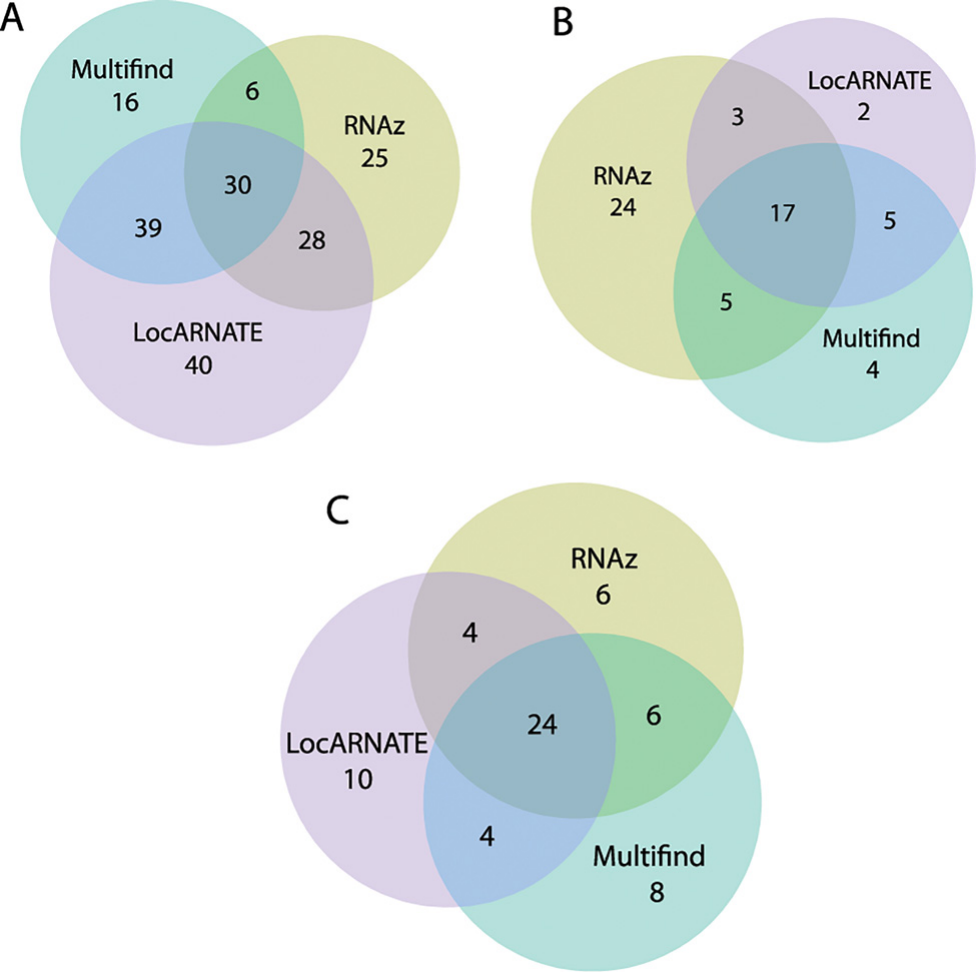
Overlap of known ncRNAs discovered by three methods. (A) The Venn diagram of the known ncRNAs predicted on (A) *S*. *cerevisiae* genome among the top 500 candidates by each method. (B) *E*. *coli* genome among the top 500 candidates by each method. (C) *S*. *coelicolor* genome among the top 100 candidates by each method.

**Fig 14:**
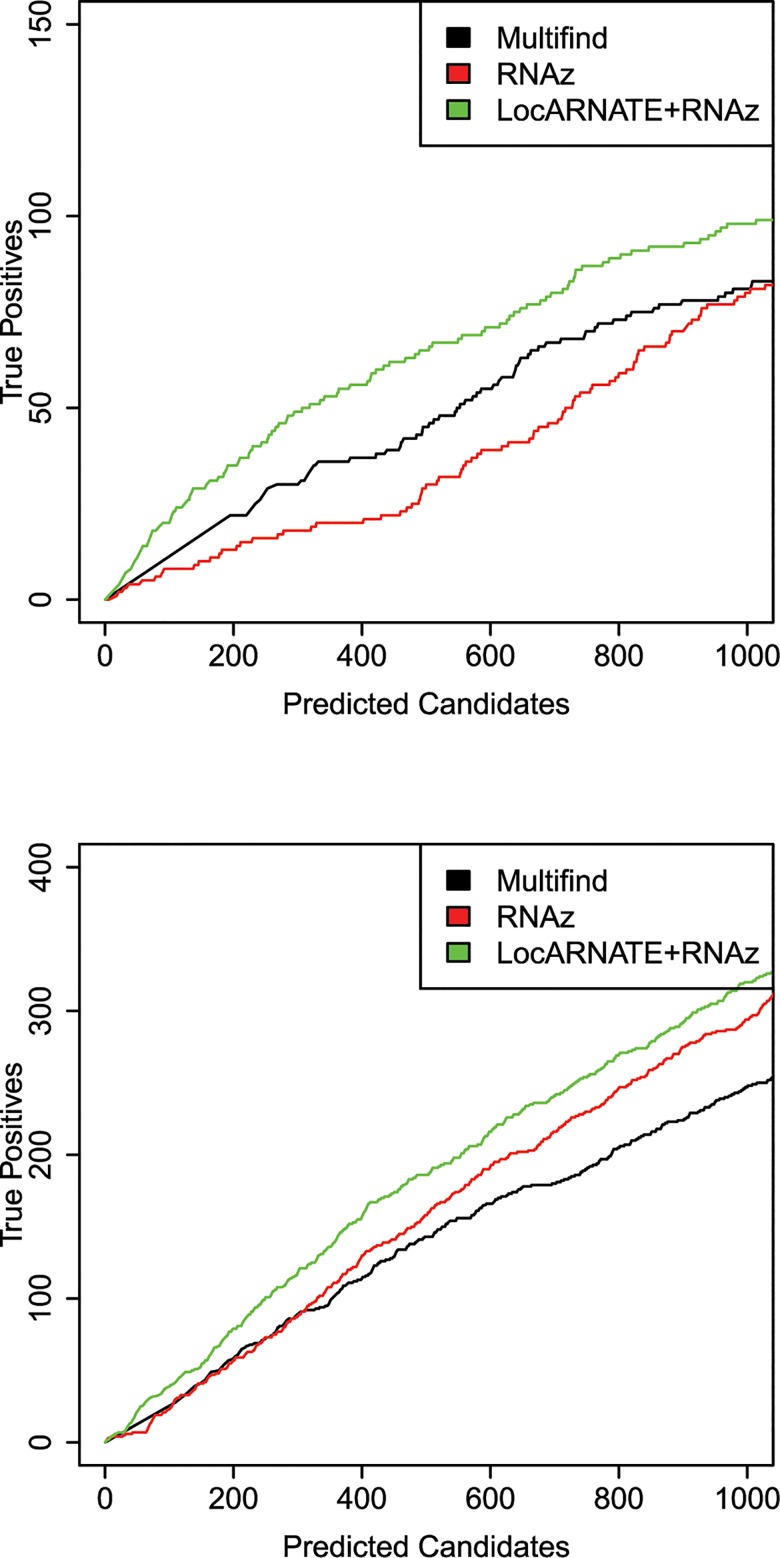
Benchmarks for ncRNA discovery in yeast. (A) True positives versus total number of predicted candidates for the *S*. *cerevisiae* genome on low similarity (S>0.3) alignment windows. (B) True positives versus total number of predicted candidates curve for the *S*. *cerevisiae* genome on high similarity (S<0.3) alignment windows.

The plots for the benchmarks on these three genomes do not show an advantage for Multifind or LocARNATE+RNAz when all windows are considered (Figs 10–12). Further analysis, however, showed that Multifind, LocARNATE+RNAz and RNAz discover different known ncRNAs (Fig 13). Table 1 also shows that the true positives in the most probable candidates predicted by Multifind, LocARNATE+RNAz and RNAz have different mean sequence similarities. Multifind and LocARNATE+RNAz tend to predict alignments with high Shannon entropy to be ncRNA. This suggests Multifind and LocARNATE+RNAz have an advantage for prediction on low similarity windows, which corresponds to the benchmarks on the Rfam sequences. To test this hypothesis, all the windows of the yeast alignment were divided into low similarity (S<0.3) and high similarity (S>0.3) categories. True candidates versus predicted candidate curves were plotted on these two sets of windows separately (Fig 14). Results showed, for high similarity windows, RNAz shows a clear advantage, but for low similarity windows, Multifind and LocARNATE+RNAz performed better.

**Table 1:** Shannon entropy of the known ncRNAs among top candidates predicted by Multifind, LocARNATE+RNAz and RNAz.

	mean Shannon entropy		
	Discovered by Multifind	Discovered by RNAz	Discovered by Locarnate+RNAz
*E*. *coli* (TP in top 500)	0.26±0.16	0.17±0.14	0.28±0.14
*S*. *coelicolor* (TP in top 100)	0.34±0.13	0.34±0.13	0.35±0.12
*S*. *cerevasiae* (TP in top 500)	0.22±0.15	0.12±0.13	0.22±0.15

### Time Consumption

Multifind inherently scales O(N^6^M) for M sequences of length N. In spite of the use of heuristics to accelerate the calculation [10, 43], its time consumption was higher than for RNAz, which scales O(N^3^) and LocARNATE, which empirically scales O(N^4^M^2^). To quantify the time usage of Multifind, LocARNATE and RNAz, two benchmarks were done on 100 randomly-chosen alignments from the Rfam training set and 100 randomly-chosen alignments from the yeast data set. The results (Table 2) showed Multifind consumes more time on the Rfam training set than LocARNATE+RNAz and RNAz. But on the yeast data set, Multifind consumes about as much time as LocARNATE+RNAz. The difference in time required by Multifind on two data sets of about the same size was because of the alignment envelope [43] that constrains Multifind’s alignment space based on a Hidden Markov Model posterior alignment probability between sequence pairs. Sequence pairs in the yeast data set have much higher percentage identity, hence a much more concentrated posterior alignment probability and a narrower alignment envelope.

**Table 2:** Time consumption of Multifind, LocARNATE+RNAz and RNAz on 100 Rfam alignments and 100 yeast alignments on a single core of an Intel Xeon CPU E5450 @ 3.00GHz.

Methods	Multifind	LocARNATE+RNAz	RNAz
Rfam alignments	7h:38min	45min	3min
yeast alignments	1h:8min	44min	4min

## Discussion

A ncRNA detection method called Multifind, based on Multilign, was developed. The benchmarks on alignments extracted from Rfam show that Multifind performed better overall on Rfam testing sets than RNAz and LocARNATE+RNAz. Its advantage is more obvious on low-identity alignments, where it performs better than RNAz and similarly to LocARNATE+RNAz. Benchmarks on genomes, however, showed that RNAz is more effective overall in detecting known ncRNAs in genome alignments. Further analysis showed Multifind and LocARNATE were more sensitive at discovering known ncRNAs in low-similarity genome alignment regions, and Multifind, LocARNATE+RNAz and RNAz independently predict a significant number of non-overlapping candidates. The latter point was also shown by the study of Vockenhuber et al. [13] where Dynalign and RNAz were compared.

The above results suggested there is no single best method for ncRNA discovery in genomes; different methods independently provide different correct candidates (Fig 13). Multifind, RNAz and LocARNATE+RNAz are applicable on genome-alignment regions with different sequence similarities. Multifind and LocARNATE+RNAz apply better on regions with low similarity, and RNAz applies better on regions with high similarity. The benchmark showed, for the yeast genome alignment, an average Shannon entropy of 0.25 would be a reasonable threshold for applying different methods. Interestingly, among the 74,484 windows of yeast alignment, 88% (65,886) are low similarity and therefore only 12% (8,597) are high similarity. But low-similarity windows include only 47% of all the known ncRNAs, showing an enrichment of known ncRNAs in high-similarity windows. Therefore, it can be argued that, because it is more convenient to search for functional elements in highly conserved regions in genomes, there are possibly more unknown ncRNAs in low-similarity genome-alignment regions. Also, discovering ncRNAs in low-similarity alignments presents a technical barrier that cannot be overcome without paying a computational price. Multifind is therefore a beneficial tool in finding ncRNAs, and it can be a complement to other methods like RNAz and LocARNATE+RNAz.

For benchmarks on genomes, the three methods are all applied on sliding windows with the size of 100 nucleotides. Performing *de novo* ncRNA discovery on windows is a common practice to limit the computational cost for scanning [44, 45]. This practice does not necessarily overlook ncRNA longer than the window size. Uzilov et al. demonstrated, in the sliding-window method, long ncRNA can be found multiple times because there are a number of conserved secondary structural elements that are shorter than the window size [10].

In addition to using Multilign, Multifind introduces an additional discriminating feature that has not been previously used for ncRNA discovery, ensemble defect. This feature quantifies the compactness the folding space of a putative ncRNA. An SVM trained with this feature can outperform a method trained without this feature for some data sets (Figs 4 and 8). This supports the hypothesis that ncRNA sequences will fold specifically to up to only a few structures.

Multifind is available as part of the RNAstructure [22] package (http://rna.urmc.rochester.edu/RNAstructure.html). It is provided under the GNU public license.

## Conclusions

A new method, Multifind, that identifies conserved ncRNA from input unaligned sequences was developed. Benchmarks on Rfam datasets showed Multifind performs better than RNAz and LocARNATE+RNAz, especially on dissimilar sequences. Benchmarks on genomes also showed Multifind and LocARNATE+RNAz are more successful than RNAz on alignments of dissimilar sequences. Because each of the three methods finds a distinct subset of the known ncRNAs, a comprehensive search for ncRNAs would use all three tools.

## Supporting Information

S1 Fig ΔG° Z Scores. (A) and ensemble defect Z Scores (B) predicted using the SVM compared with calculated from shuffling 1000 times on 1000 randomly generated sequences. The correlation between predicted ΔG° Z Score and sampled ΔG° Z Score was R = 0.998. The correlation between predicted ensemble defect Z Score and sampled ensemble defect Z Score was R = 0.999.
(EPS)

S1 Table The length distribution of the ncRNAs acquired from NCBI, Rfam 10.1 (DOC)

S2 Table The distribution of windows according to the percentage of the nucleotides in them that overlaps with a ncRNA. (DOC)

S1 File Positive Training Set. This file contains the complete set of positive training data. A key indicates the distinct subset of alignments used for each of the four testing sets.
(ZIP)

S2 File Negative Training Set. This file contains the complete set of negative training data. A key indicates the distinct subset of alignments used for each of the four testing sets.
(ZIP)

## References

[1] Eddy SR. Non-coding RNA genes and the modern RNA world. Nat Rev Genet. 2001;2(12):919–29. 10.1038/35103511 11733745

[2] Waters LS, Storz G. Regulatory RNAs in bacteria. Cell. 2009;136(4):615–28. 10.1016/j.cell.2009.01.043 19239884 PMC3132550

[3] Cech TR, Steitz JA. The noncoding RNA revolution—trashing old rules to forge new ones. Cell. 2014;157(1):77–94. 10.1016/j.cell.2014.03.008 24679528

[4] Encode Project Consortium. An integrated encyclopedia of DNA elements in the human genome. Nature. 2012;489(7414):57–74. 10.1038/nature11247 22955616 PMC3439153

[5] Chodroff RA, Goodstadt L, Sirey TM, Oliver PL, Davies KE, Green ED, et al Long noncoding RNA genes: conservation of sequence and brain expression among diverse amniotes. Genome Biology. 2010;11(7):R72 Artn R72 10.1186/Gb-2010-11-7-R72 . 20624288 PMC2926783

[6] Rivas E, Eddy SR. Noncoding RNA gene detection using comparative sequence analysis. BMC Bioinformatics. 2001;2:8 10.1186/1471-2105-2-8 11801179 PMC64605

[7] Pedersen JS, Bejerano G, Siepel A, Rosenbloom K, Lindblad-Toh K, Lander ES, et al Identification and classification of conserved RNA secondary structures in the human genome. Plos Comput Biol. 2006;2(4):e33 10.1371/journal.pcbi.0020033 16628248 PMC1440920

[8] Washietl S, Hofacker IL, Stadler PF. Fast and reliable prediction of noncoding RNAs. Proc Natl Acad Sci U S A. 2005;102(7):2454–9. 10.1073/pnas.0409169102 15665081 PMC548974

[9] Yao Z, Weinberg Z, Ruzzo WL. CMfinder—a covariance model based RNA motif finding algorithm. Bioinformatics. 2006;22(4):445–52. 10.1093/bioinformatics/btk008 . 16357030

[10] Uzilov AV, Keegan JM, Mathews DH. Detection of non-coding RNAs on the basis of predicted secondary structure formation free energy change. BMC Bioinformatics. 2006;7:173 10.1186/1471-2105-7-173 16566836 PMC1570369

[11] Washietl S, Pedersen JS, Korbel JO, Stocsits C, Gruber AR, Hackermuller J, et al Structured RNAs in the ENCODE selected regions of the human genome. Genome Res. 2007;17(6):852–64. 10.1101/gr.5650707 17568003 PMC1891344

[12] Torarinsson E, Yao Z, Wiklund ED, Bramsen JB, Hansen C, Kjems J, et al Comparative genomics beyond sequence-based alignments: RNA structures in the ENCODE regions. Genome Res. 2008;18(2):242–51. 10.1101/gr.6887408 18096747 PMC2203622

[13] Vockenhuber MP, Sharma CM, Statt MG, Schmidt D, Xu Z, Dietrich S, et al Deep sequencing-based identification of small non-coding RNAs in Streptomyces coelicolor. RNA Biol. 2011;8(3):468–77. 21521948 10.4161/rna.8.3.14421 PMC3218513

[14] Gruber AR, Findeiss S, Washietl S, Hofacker IL, Stadler PF. RNAz 2.0: improved noncoding RNA detection. Pac Symp Biocomput. 2010;15:69–79. Epub 2009/11/13. . 19908359

[15] Mathews DH, Turner DH. Dynalign: an algorithm for finding the secondary structure common to two RNA sequences. J Mol Biol. 2002;317(2):191–203. 10.1006/jmbi.2001.5351 . 11902836

[16] Torarinsson E, Sawera M, Havgaard JH, Fredholm M, Gorodkin J. Thousands of corresponding human and mouse genomic regions unalignable in primary sequence contain common RNA structure. Genome Res. 2006;16(7):885–9. 10.1101/gr.5226606 16751343 PMC1484455

[17] Xu Z, Mathews DH. Multilign: an algorithm to predict secondary structures conserved in multiple RNA sequences. Bioinformatics. 2011;27(5):626–32. 10.1093/bioinformatics/btq726 21193521 PMC3042186

[18] Gardner PP, Daub J, Tate J, Moore BL, Osuch IH, Griffiths-Jones S, et al Rfam: Wikipedia, clans and the "decimal" release. Nucleic Acids Res. 2011;39(Database issue):D141–5. 10.1093/nar/gkq1129 21062808 PMC3013711

[19] Gardner PP, Daub J, Tate JG, Nawrocki EP, Kolbe DL, Lindgreen S, et al Rfam: updates to the RNA families database. Nucleic Acids Res. 2009;37(Database issue):D136–40. 10.1093/nar/gkn766 18953034 PMC2686503

[20] Mathews DH, Disney MD, Childs JL, Schroeder SJ, Zuker M, Turner DH. Incorporating chemical modification constraints into a dynamic programming algorithm for prediction of RNA secondary structure. Proc Nat Acad Sci USA. 2004;101(19):7287–92. 10.1073/pnas.0401799101 . 15123812 PMC409911

[21] Lu ZJ, Gloor JW, Mathews DH. Improved RNA secondary structure prediction by maximizing expected pair accuracy. RNA. 2009;15(10):1805–13. 10.1261/rna.1643609 19703939 PMC2743040

[22] Reuter JS, Mathews DH. RNAstructure: software for RNA secondary structure prediction and analysis. BMC Bioinformatics. 2010;11:129 10.1186/1471-2105-11-129 20230624 PMC2984261

[23] Turner DH, Mathews DH. NNDB: the nearest neighbor parameter database for predicting stability of nucleic acid secondary structure. Nucleic Acids Res. 2010;38(Database issue):D280–2. 10.1093/nar/gkp892 19880381 PMC2808915

[24] Xia T, SantaLucia J Jr., Burkard ME, Kierzek R, Schroeder SJ, Jiao X, et al Thermodynamic parameters for an expanded nearest-neighbor model for formation of RNA duplexes with Watson-Crick base pairs. Biochemistry. 1998;37(42):14719–35. 10.1021/bi9809425 . 9778347

[25] Mathews DH, Turner DH. Experimentally derived nearest-neighbor parameters for the stability of RNA three- and four-way multibranch loops. Biochemistry. 2002;41(3):869–80. . 11790109 10.1021/bi011441d

[26] Chang CC, Lin CJ. LIBSVM: A Library for Support Vector Machines. ACM Trans Intell Syst Technol. 2011;2(3):27 Artn 27 10.1145/1961189.1961199 .

[27] Chan CY, Ding Y. Boltzmann ensemble features of RNA secondary structures: a comparative analysis of biological RNA sequences and random shuffles. J Math Biol. 2008;56(1–2):93–105. 10.1007/s00285-007-0129-z . 17909813

[28] Zadeh JN, Wolfe BR, Pierce NA. Nucleic acid sequence design via efficient ensemble defect optimization. J Comput Chem. 2011;32(3):439–52. 10.1002/jcc.21633 . 20717905

[29] Gruber AR, Bernhart SH, Hofacker IL, Washietl S. Strategies for measuring evolutionary conservation of RNA secondary structures. BMC Bioinformatics. 2008;9(1):122 10.1186/1471-2105-9-122 18302738 PMC2335298

[30] Larkin MA, Blackshields G, Brown NP, Chenna R, McGettigan PA, McWilliam H, et al Clustal W and Clustal X version 2.0. Bioinformatics. 2007;23(21):2947–8. 10.1093/bioinformatics/btm404 . 17846036

[31] Pruitt KD, Brown GR, Hiatt SM, Thibaud-Nissen FA, Ermolaeva O, Farrell CM, et al RefSeq: an update on mammalian reference sequences. Nucleic Acids Res. 2014;42(D1):D756–D63. 24259432 10.1093/nar/gkt1114 PMC3965018

[32] Darling AE, Mau B, Perna NT. progressiveMauve: multiple genome alignment with gene gain, loss and rearrangement. PLoS One. 2010;5(6):e11147 10.1371/journal.pone.0011147 20593022 PMC2892488

[33] Kent WJ, Sugnet CW, Furey TS, Roskin KM, Pringle TH, Zahler AM, et al The human genome browser at UCSC. Genome Res. 2002;12(6):996–1006. 12045153 10.1101/gr.229102 PMC186604

[34] Riley M, Abe T, Arnaud MB, Berlyn MKB, Blattner FR, Chaudhuri RR, et al Escherichia coli K-12: a cooperatively developed annotation snapshot—2005. Nucleic Acids Res. 2006;34(1):1 16397293 10.1093/nar/gkj405 PMC1325200

[35] Bentley SD, Chater KF, Cerdeno-Tarraga AM, Challis GL, Thomson NR, James KD, et al Complete genome sequence of the model actinomycete Streptomyces coelicolor A3(2). Nature. 2002;417(6885):141–7. 10.1038/417141a . 12000953

[36] Goffeau A, Barrell BG, Bussey H, Davis RW, Dujon B, Feldmann H, et al Life with 6000 genes. Science. 1996;274(5287):546, 63–7. . 8849441 10.1126/science.274.5287.546

[37] Deng W, Liou SR, Plunkett G III, Mayhew GF, Rose DJ, Burland V, et al Comparative Genomics of Salmonellaenterica Serovar Typhi Strains Ty2 and CT18. J Bacteriol. 2003;185(7):2330 12644504 10.1128/JB.185.7.2330-2337.2003 PMC151493

[38] Holt KE, Thomson NR, Wain J, Langridge GC, Hasan R, Bhutta ZA, et al Pseudogene accumulation in the evolutionary histories of Salmonella enterica serovars Paratyphi A and Typhi. BMC Genomics. 2009;10(1):36 10.1186/1471-2164-10-36 19159446 PMC2658671

[39] Fouts DE, Tyler HL, DeBoy RT, Daugherty S, Ren Q, Badger JH, et al Complete genome sequence of the N2-fixing broad host range endophyte Klebsiella pneumoniae 342 and virulence predictions verified in mice. PLoS Genet. 2008;4(7):e1000141 10.1371/journal.pgen.1000141 18654632 PMC2453333

[40] Ohnishi Y, Ishikawa J, Hara H, Suzuki H, Ikenoya M, Ikeda H, et al Genome sequence of the streptomycin-producing microorganism Streptomyces griseus IFO 13350. J Bacteriol. 2008;190(11):4050–60. 10.1128/JB.00204-08 18375553 PMC2395044

[41] Fujita PA, Rhead B, Zweig AS, Hinrichs AS, Karolchik D, Cline MS, et al The UCSC Genome Browser database: update 2011. Nucleic Acids Res. 2011;39(Database issue):D876–82. 10.1093/nar/gkq963 20959295 PMC3242726

[42] Pruitt KD, Tatusova T, Klimke W, Maglott DR. NCBI Reference Sequences: current status, policy and new initiatives. Nucleic Acids Res. 2009;37(Database issue):D32–6. 10.1093/nar/gkn721 18927115 PMC2686572

[43] Harmanci AO, Sharma G, Mathews DH. Efficient pairwise RNA structure prediction using probabilistic alignment constraints in Dynalign. BMC Bioinformatics. 2007;8(1):130 Artn 130 10.1186/1471-2105-8-130 . 17445273 PMC1868766

[44] Stark A, Lin MF, Kheradpour P, Pedersen JS, Parts L, Carlson JW, et al Discovery of functional elements in 12 Drosophila genomes using evolutionary signatures. Nature. 2007;450(7167):219–32. 10.1038/nature06340 17994088 PMC2474711

[45] Washietl S, Hofacker IL, Lukasser M, Huttenhofer A, Stadler PF. Mapping of conserved RNA secondary structures predicts thousands of functional noncoding RNAs in the human genome. Nat Biotechnol. 2005;23(11):1383–90. 10.1038/nbt1144 . 16273071

