# Supplementary material for: Discovery of Novel ncRNA Sequences in Multiple Genome Alignments on the Basis of Conserved and Stable Secondary Structures
Source: PLoS One. 2015 Jun 15;10(6):e0130200. doi: 10.1371/journal.pone.0130200 (PMC4468099; doi:10.1371/journal.pone.0130200)
Supplement: S1 Table — (DOC) [file pone.0130200.s002.doc]

| Length (nt) | <60 | 60-80 | 80-100 | 100-120 | 120-140 | 140-200 | 200+ |
| --- | --- | --- | --- | --- | --- | --- | --- |
| Distribution of ncRNAs in the *E. coli* genome | 5.6% | 39.5% | 14.4% | 18.1% | 8.8% | 13.5% | 11.6% |
| Distribution of ncRNAs in the *S. coelicolor* genome | 60.7% | 17.7% | 8.9% | 4.3% | 3.4% | 4.9% | 2.9% |
| Distribution of ncRNAs in the *S. cerevasiae* genome | 0% | 55% | 20.9% | 13.4% | 3.4% | 6.9% | 8.1% |
