# Supplementary material for: Discovery of Novel ncRNA Sequences in Multiple Genome Alignments on the Basis of Conserved and Stable Secondary Structures
Source: PLoS One. 2015 Jun 15;10(6):e0130200. doi: 10.1371/journal.pone.0130200 (PMC4468099; doi:10.1371/journal.pone.0130200)
Supplement: S2 Table — (DOC) [file pone.0130200.s003.doc]

| Percentage of nucleotides that overlap with a ncRNA(%) | 0-20 | 20-40 | 40-60 | 60-80 | 80-100 |
| --- | --- | --- | --- | --- | --- |
| Distribution of windows from *E. coli* genome alignments | 0% | 17.5% | 29.6% | 33.9% | 18.9% |
| Distribution of windows from *S. coelicolor* genome alignments | 1.8% | 35.9% | 32.2% | 18.9% | 11.2% |
| Distribution of windows from *S. cerevasiae* genome alignments | 0% | 4.8% | 13.7% | 25.8% | 55.7% |
