# Supplementary material for: Discovery of Novel ncRNA Sequences in Multiple Genome Alignments on the Basis of Conserved and Stable Secondary Structures
Source: PLoS One. 2015 Jun 15;10(6):e0130200. doi: 10.1371/journal.pone.0130200 (PMC4468099; doi:10.1371/journal.pone.0130200)
Supplement: S1 File — This file contains the complete set of positive training data. A key indicates the distinct subset of alignments used for each of the four testing sets. (ZIP) [file pone.0130200.s004.zip › S1_File/description of the data.docx]

The positive data set is in the “positive” training set file. The negative data set is in the “negative” training set file. Every file’s name includes a “RFXXXXX” part that indicates the Rfam family to which it belongs. In the paper, the results of four tests were presented. The families that are included in the testing sets of the four benchmarks are listed below. For each test, the remaining families were used for training.

1^st^ benchmark:

RF00031

RF00020

RF00648

RF00559

RF00019

RF00647

RF00130

RF00257

RF00263

RF01118

RF00057

RF00892

RF00190

RF01057

2^nd^ benchmark:

RF00515

RF00066

RF00379

RF00654

RF01051

RF00442

RF00008

RF00238

RF00050

RF00668

RF01067

RF01061

RF00144

RF00460

3^rd^ benchmark:

RF00468

RF00239

RF00651

RF00051

RF00391

RF00646

RF01116

RF00485

RF00667

RF00736

RF00103

RF01394

RF00906

RF00504

RF00685

RF00029

RF00080

4^th^ benchmark:

RF00645

RF00558

RF00015

RF01059

RF00053

RF00672

RF00027

RF00566

RF00185

RF01045

RF00246

RF00657

RF00094

RF00525

RF00490

RF00617

RF00464
